# Does the Proximity of Meals to Bedtime Influence the Sleep of Young Adults? A Cross-Sectional Survey of University Students

**DOI:** 10.3390/ijerph17082677

**Published:** 2020-04-14

**Authors:** Nikola Chung, Yu Sun Bin, Peter A. Cistulli, Chin Moi Chow

**Affiliations:** 1 Sydney Medical School, University of Sydney, Sydney 2006, Australia; nchu9396@uni.sydney.edu.au (N.C.); peter.cistulli@sydney.edu.au (P.A.C.); 2 Sleep Research Group, Charles Perkins Centre, University of Sydney, Sydney 2006, Australia; chin-moi.chow@sydney.edu.au; 3 Sydney School of Health Sciences, University of Sydney, Sydney 2006, Australia

**Keywords:** food intake, meal time, sleep disturbance, sleep quality, sleep hygiene, eating behaviours

## Abstract

Avoiding food before bedtime is a widely accepted sleep hygiene practice, yet few studies have assessed meal timing as a risk factor for disrupted sleep. This study examined the relationship between evening meal timing and sleep quality in young adults. A total of *N* = 793 participants (26% male) aged between 18 and 29 years responded to an online survey, which captured sociodemographic information, lifestyle variables, and sleep characteristics. Meal timing was defined as meals more than 3 h before or within 3 h of bedtime. The outcomes were as follows: one or more nocturnal awakenings, sleep onset latency of >30 min, and sleep duration of ≤6 h. Logistic regression analyses showed that eating within 3 h of bedtime was positively associated with nocturnal awakening (OR = 1.61, 95% CI = 1.15–2.27) but not long sleep onset latency (1.24; 0.89–1.73) or short sleep duration (0.79; 0.49–1.26). The relationship remained significant after adjusting for potential confounders of ethnicity and body mass index (OR = 1.43, 95% CI = 1.00–2.04). Meal timing appears to be a modifiable risk factor for nocturnal awakenings and disrupted sleep. However, this is a preliminary cross-sectional study and highlights the need for additional research on the influence of the timing of food intake on sleep.

## 1. Introduction

University students are vulnerable to poor sleep due to the society-wide increase in technology use [1], the increased rates of substance abuse [2] and binge drinking in this age group [3], and the perceived stress [4] and irregular sleep schedules [5] related to academic and social commitments. This is problematic given that epidemiological studies have consistently linked poor chronic sleep patterns to major health concerns such as weight gain and obesity [6], diabetes [7,8,9], hypertension [8,9], and cardiovascular disease and stroke [8,9,10]. More acutely, poor sleep quality is associated with increased risks for mental health problems [4,9,11] as well as decreased concentration and performance [12].

Many modifiable risk factors for poor sleep are behavioural in nature. These include cigarette smoking [13], caffeine intake [14], alcohol consumption [13], and poor diet [15]. In contrast, physical activity [16] and regularity in the timing of lifestyle behaviours have been associated with improved sleep [17].

The timing of the evening meal can vary considerably due to work and lifestyle demands. Avoiding meals close to bedtime is a common piece of sleep hygiene advice. It is thought that meals close to sleep onset contribute to poor sleep quality through gastrointestinal discomfort, heartburn, and reflux [18,19]. Further, the metabolism of food demonstrates circadian rhythmicity and digestion at night is less efficient than during the day [20]. However, how meal timing affects sleep is not well researched. To our knowledge, only one study has examined the effect of meal timing on sleep: Afaghi and colleagues showed that a high glycaemic index (GI) meal ingested 4 h before bedtime promoted the onset of sleep more effectively than the same meal ingested 1 h before bedtime [21]. The present study sought to examine the association between eating within 3 h of bedtime on self-reported sleep parameters.

## 2. Materials and Methods

### 2.1. Participants and Procedures

Participants in this study were university students from the University of Sydney and Macquarie University, in Sydney, Australia. Students and staff registered at the two universities at the time of the survey were invited via an email link to participate in an anonymised online survey aimed at investigating links between the home sleep environment and self-reported sleep. The survey contained a range of questions about the participants’ sleep environment, sleep, other health behaviours, and demographic information. The data were collected over a period of two weeks in early September 2012. The survey was approved by the University of Sydney’s Human Research Ethics Committee (Protocol Number: 14209). Students consented to participate by completing the survey. Upon completion of the survey, participants were eligible to enter a draw for one of ten $50 prizes or a single $500 prize.

Overall, 1023 participants completed the survey. The survey data were checked for the validity of answers, resulting in the removal of three participants due to incomplete responses. Participants aged 30 years and over (*n* = 227) were excluded from the data to restrict the current study to a homogeneous sample of only young adults. Therefore, the total sample size in this study was *N* = 793 (587 females and 206 males).

### 2.2. Measures

Evening meal timing was the main exposure of interest, and this was queried as the typical number of hours between the final meal of the day and bedtime (≤1 h/>1 to 2 h/>2–3 h/>3 to 4 h/>4 h before bedtime). This variable was dichotomised into “≤3 h before bedtime” and “>3 h before bedtime” according to the sleep hygiene advice that evening meals should be avoided in the 2–3 h before bed.

Self-reported sleep was the main outcome and included the following: usual bedtime and usual wake time, from which sleep duration (in hours) was calculated; number of nocturnal awakenings; and usual sleep onset latency (SOL, in minutes). Sleep duration was categorised into “6 h or less” or “7 h or more”. Nocturnal awakenings were categorised into “none” or “one or more”. SOL was categorised into “30 min or less” or “more than 30 min”.

Demographic covariates included participant age (18–21 years/22–25 years/26–29 years), gender (male/female), and ethnic background (Caucasian/Asian/other). Body mass index was calculated using self-reported weight (kg) divided by self-reported height squared (m^2^) and categorised into underweight (<18.5 kg/m^2^), normal (18.5–24.9 kg/m^2^), overweight (25.0–29.9 kg/m^2^), and obese (>30.0 kg/m^2^).

Health behaviours, such as smoking status (current smokers/non-smokers), self-rated physical activity (sedentary/moderately active/vigorously active), daily caffeine consumption (none/1–2 drinks/3 or more drinks), caffeine consumption at bedtime (none/1+ drinks), daily alcohol consumption (none/any), alcohol intake at bedtime (none/1+ drinks), and total beverages consumed at bedtime (none/one/two or more), were also considered as potential covariates. Caffeine consumption was derived from questions about the number of coffee, tea, and energy drinks consumed daily and at bedtime. Similarly, alcohol intake was defined as number of glasses of beer, wine, or other alcoholic drinks consumed daily and at bedtime. Total beverages consumed at bedtime was calculated from the number of cups/glasses of caffeinated beverages, alcoholic beverages, decaffeinated coffee, carbonated drinks, and chocolate- and milk-based drinks combined. We note that since the survey did not distinguish between different types of tea, we included tea in the consideration of caffeinated beverages. Chocolate-containing drinks were considered not to contain caffeine because the amount of caffeine in typical drinks is about one-tenth that in a typical cup of coffee.

The survey measures used to collect data for this study can be seen in Appendix A.

### 2.3. Data Analysis

Chi-square tests were used to identify significant associations between the sleep parameters (nocturnal awakening, sleep onset latency, and sleep duration) and the sociodemographic and health behaviour variables. Logistic regression models were used to describe the association between meal timing and each of the sleep parameters, with and without control for potential confounders. Sociodemographic and behavioural risk factors that were found to be significantly associated with both meal timing and the relevant sleep parameter were included in the logistic regressions as potential confounders. All covariates were entered as categorical variables in the adjusted models. Sensitivity analysis additionally controlled for the influence of caffeine and alcohol consumption at bedtime and beverage intake at bedtime and examined the influence of different meal timing cut-off points on the results (the results are presented in Appendix B). IBM SPSS Statistics for Macintosh Version 24.0. (IBM Corp., Armonk, NY, USA) was used for all analyses. A significance level of 0.05 was used throughout.

## 3. Results

### 3.1. Participants

Table 1 describes the sociodemographic and health behaviours of the sample. The majority of participants were female (74.0%), Caucasian (63.6%), aged 21 and below (55.5%), and non-smokers (95.2%). Most (61.2%) reported themselves as moderately active, and 72.9% had a normal BMI. Less than a quarter of participants consumed alcohol on an average day (23%), and 11.6% would consume alcohol at bedtime. Consumption of caffeinated beverages was more common than the consumption of alcohol, with 77.1% having one or more caffeinated drinks on an average day and 35.4% having a caffeinated drink at bedtime. More than half of participants (58.9%) reported drinking at bedtime.

### 3.2. Meal Timing by Sociodemographic and Behavioural Risk Factors

More than half of students (53.7%) ate within the 3 h before bedtime. Table 1 shows the distribution of meal timing by the sociodemographic and health behaviours of the study sample. There were no significant gender or age differences in meal timing, although Asian participants were less likely to report later meal timings compared to participants of other ethnicities (37.6% vs. 53.7% overall). BMI appeared to be associated with later meal timing in a dose-dependent manner, with increasing BMI linked to increasing rates of eating within 3 h of bedtime. Physical activity was also associated with later meal timing, with larger proportions of physically active students reporting meals within 3 h of sleep. Cigarette smoking and evening intake of caffeine was not significantly associated with evening meal timing, but those with evening alcohol intake were also more likely to report eating close to bedtime compared to those who did not drink alcohol in the evenings (68.5% vs. 55.2%).

### 3.3. Sleep and Sociodemographic and Behavioural Risk Factors

Nocturnal awakening was reported by 24.1% of participants, long SOL by 23.5%, and ≤6 h sleep durations by 9.6%. Table 1 shows the distribution of sleep outcomes by sociodemographic and behavioural risk factors. There were significant differences by age on sleep, most notably for nocturnal awakenings and short sleep duration. The youngest age group (18–21-year olds) was least likely to experience nocturnal awakenings (19.8%), with nocturnal awakenings increasing with age (e.g., 30.3% in the 26–29-year-old age group). However, the younger age groups were more likely to report long SOL and short sleep durations of <7 h than the oldest participants.

Women were more likely to report nocturnal awakenings than men (26.6% vs. 17.0%) and to report long SOL (25.9% vs. 16.5%), but there was no difference in sleep duration between genders. Significant differences were seen between students of different ethnicities on all sleep parameters.

Individuals with obesity appeared to experience more nocturnal awakenings than underweight individuals, whilst normal and overweight individuals had similar rates of nocturnal awakening. However, individuals with obesity also appeared to have lower rates of long SOL and short sleep durations than those with lower BMI. Current smokers were more likely to experience a longer SOL than non-smokers, but there was no difference in nocturnal awakenings or average sleep duration for smokers compared to non-smokers. Level of physical activity was not significantly associated with any of the sleep parameters, although the small number who engaged in vigorous physical activity appeared to have fewer nocturnal awakenings and were less likely to report short sleep durations. Surprisingly, neither evening alcohol intake nor evening caffeine intake was significantly associated with any of the sleep parameters.

### 3.4. Meal Timing and Sleep

Those who ate within 3 h of bedtime were more likely to experience nocturnal awakenings (27.8%) than those who ate more than 3 h before bedtime (19.2%), but meal timing was not significantly associated with either SOL or sleep duration (Table 1).

Table 2 shows the relationship between evening meal timing and the sleep parameters, with and without adjustment for covariates. Evening meal timing within 3 h of sleep was significantly associated with nocturnal awakening, but not long sleep onset latency or short sleep duration (Table 2).

Ethnicity and BMI were associated with both meal timing and nocturnal awakenings in the chi-square analysis and were therefore included in the adjusted regression model for nocturnal awakenings. After adjusting for these confounders, nocturnal awakening was still elevated in those who consumed their final evening meal within 3 h of bedtime (*p* = 0.05).

Ethnicity was the only variable associated with both meal timing and SOL, and with short sleep duration, and hence, ethnicity was the only variable included in the adjusted analysis for SOL and short sleep duration. Meal timing was not associated with SOL or sleep duration in both the adjusted and unadjusted models (Table 2).

## 4. Discussion

Our results show that eating within 3 h of bedtime is associated with a ~40% increase in the odds of nocturnal awakening after accounting for ethnicity and BMI. This is consistent with the commonplace belief and sleep hygiene advice that one should not eat close to bedtime and suggests that we may be able to improve sleep by manipulating meal timing. However, meal timing was not associated with SOL or sleep duration. Sensitivity analysis with different cut-off points for meal timing—within 2 h of bedtime and within 4 h of bedtime—revealed similar results (see Appendix B).

It appears that the timing of the evening meal relative to sleep time is a specific contributor to nocturnal awakenings and does not affect sleep latency or sleep duration. It is possible that later food and fluid intake is associated with nocturnal awakening through nocturia. However, we did not find an association between caffeine and alcohol intake at bedtime with nocturnal awakenings in this population, nor was there an association between total beverages ingested at bedtime with nocturnal awakenings (see Appendix B). Instead, normal digestion and gastroesophageal reflux may be responsible for the association between evening meal timing and nocturnal awakenings. Reflux has been shown to be associated with eating close to bedtime as well as disrupted sleep [18,19], and gastric emptying time is estimated to be between 2 and 4 h after meal ingestion [22]. 

Although many studies have examined the influence of meal timing as a consequence of circadian misalignment [20] and as a contributor to circadian desynchrony resulting in metabolic dysfunction [23], few studies have assessed how evening meal timing specifically affects sleep in a healthy population. A previous study of 300 patients with sleep apnoea found that later meal timing was associated with longer objectively measured sleep latency, higher apnoea-hypopnoea index, and poorer subjective sleep quality [24]. However, in that study, the proximity of the final meal relative to bedtime was not evaluated. Rather, those with dinner times later than 8 p.m. were compared to those with earlier dinner times. The longer sleep latency observed in that study was small, in the order of 0.1 min on polysomnography, and thus may not be well captured by self-reported measures of sleep latency in the current study.

To our knowledge, meal timing and its relationship with sleep latency and sleep duration has not been previously explored. Previous studies have found that the composition of the evening meal influences sleep latency. Afaghi and colleagues reported that the consumption of a high glycaemic index (GI) evening meal was associated with a shorter sleep onset latency [21], while Crispim et al. found that high-calorie and high-fat meals within 60 min of sleep were correlated with longer sleep latency, decreased sleep efficiency, and increased wake after sleep onset [25]. We did not measure the composition of meals, but it is possible that later meal timing reflects meals of a different macronutrient composition than earlier meals [26,27] and that controlling for glycaemic index and other indicators of meal composition may reveal an underlying relationship between meal timing and sleep. For instance, macronutrients such as the amino acid tryptophan and micronutrients such as group B vitamins are thought to promote sleep as both are required for the production of serotonin and melatonin [28], the neurotransmitters involved in sleep regulation.

The absence of a relationship between meal timing and sleep duration is less surprising because sleep duration is predominately determined by the time since last sleep and social factors such as hours of employment or study, which influence habitual bed- and wake-times [29]. Habitual sleep duration has, however, been associated with meal composition, albeit inconsistently: both increased [30] and decreased fat intake [31] and low- [31] and high-carbohydrate [32] diets have been found in those reporting short sleep durations. Clearly, the influence of meal composition, both in terms of macro- and micronutrients, meal size, and meal timing, must be explored in future studies in order to improve our understanding of the relationship between diet and sleep.

The present study has several limitations. Firstly, this study was conducted using data collected for a different purpose. The primary objective of the original survey was to investigate if the home sleep environment and diet and exercise pattern have a relationship with the quality of sleep in university students. Thus, this study did not assess food-related variables, such as the size or composition of the final evening meal, nor the influence of snacking after dinner, i.e., the timing of last food intake prior to sleep. It is possible that the timing of last food intake is actually later in those with earlier evening meal times due to a longer opportunity to snack after dinner and decreasing satiety since dinner. However, if that were true, then any effects of meal timing on sleep would likely be biased towards the null, i.e., would make our results a conservative estimate of the influence of meal timing.

Potential confounders, such as shift work, which influence both meal timing and sleep, were not assessed by the survey and should be included in future studies. Causal relationships cannot be elicited from this cross-sectional study; therefore, prospective studies that manipulate meal timing are required to address the direction of causality. It is possible that those with nocturnal awakenings are more likely to have later meal times, but there is no clear reason to suppose this hypothesis is more likely than later meal times causing more nocturnal sleep disturbances. Thirdly, while there were a large number of participants in this study, we relied on self-reported data, which are subjective in nature. Objective sleep measures, such as polysomnography or actigraphy, would be helpful in future studies as an adjunct to self-report. Participants were self-selected into the study and selection and response biases may have influenced the results of the study. The convenience sample was certainly not representative of university students. However, participants were blind to the hypothesis of the current study, potentially reducing bias in the estimate of the association between meal timing and sleep. Lastly, we cannot rule out the potential for false positive findings in using multiple statistical comparisons, and as with every study, the findings need to be replicated.

The strengths of this study include the sociodemographic and lifestyle confounders that were examined simultaneously. We were able to examine the influence of meal timing on multiple self-reported sleep parameters, in contrast to many previous studies that only use single measures, such as sleep duration, as a proxy for poor sleep. By examining the effect of meal timing on multiple sleep parameters simultaneously, we were able to show that nocturnal awakenings may be more vulnerable to changing meal timing than other aspects of sleep. Currently, there are few studies that have examined the link between meal timing and sleep, and this is the key strength of this study given the growing interest in identifying modifiable risk factors for poor sleep.

Loading of energy and nutrients is not expected at night time, during the sleep phase of the activity–rest cycle. Manipulating meal timing uncouples peripheral clocks from the main circadian rhythm and alters glucose metabolism [33,34], implicating meal timing as a risk factor for cardiovascular [35,36,37] and metabolic disease [34,38]. Eating close to sleep predicts a higher total daily caloric intake in healthy adults, potentially increasing the risk of weight gain [27]. Our study provides an additional link; that meal timing may influence sleep quality in addition to disturbing circadian rhythmicity. The extent to which the current findings can be used to improve sleep in at-risk groups, such as university students and shift workers, is not well known. Altering meal timing appears to result in more effective weight loss in overweight/obese individuals [39], whilst earlier meal timing in conjunction with a regimen of light exposure and earlier sleep- and waketimes appear to be beneficial to cognition and mental health [40]. Thus, given the already known links between poor sleep and health, future studies should aim to further investigate the effectiveness of manipulating meal timing to improve sleep as a way of reducing unfavourable health outcomes.

## 5. Conclusions

In summary, meal timing is a potential risk factor for nocturnal awakenings and poor sleep quality. While this is a preliminary study, meal timing is modifiable. These findings suggest that targeting meal timing may help to improve sleep quality and potentially assist in preventing poor health outcomes. The efficacy and impact of altering meal timing on sleep is unknown; therefore, more research is required to further disentangle the complex relationship between nutrition and sleep health.

## Figures and Tables

**Table 1:** Meal timing and sleep by sociodemographic and behavioural risk factors in students aged 18–29 years (*N* = 793).

Characteristics	Total Sample *N* = 793 *n* (col%)	Meal Timing <3 h before Bed *n* = 450 (row%)	One or More Nocturnal Awakenings *n* = 191 (row%)	Sleep Onset Latency >30 min *n* = 186 (row%)	Sleep Duration ≤6 h *n* = 76 (row%)
*Meal timing*					
>3 h before bedtime	343 (43.2)	-	**19.2**	21.3	10.8
≤3 h before bedtime	450 (56.8)	-	**27.8**	25.1	8.7
*Age*					
18–21 years	440 (55.5)	53.4	**19.8**	25.5	**10.7**
22–25 years	221 (27.9)	61.5	**29.0**	22.2	**10.9**
26–29 years	132 (16.6)	59.8	**30.3**	18.9	**3.8**
*Gender*					
Female	587 (74.0)	55.7	**26.6**	**25.9**	8.9
Male	206 (26.0)	59.7	**17.0**	**16.5**	11.7
*Ethnicity*					
Caucasian	504 (63.6)	**63.9**	**25.6**	**26.4**	**6.2**
Asian	234 (29.5)	**37.6**	**18.8**	**16.7**	**17.1**
Other	55 (7.0)	**72.7**	**32.7**	**25.5**	**9.1**
*Body mass index (kg/m^2^)*					
Underweight	80 (10.1)	**56.3**	**25.0**	20.0	12.5
Normal	570 (71.9)	**54.9**	**23.3**	23.7	10.0
Overweight	113 (14.2)	**60.2**	**19.5**	26.5	7.1
Obese	30 (3.8)	**80.0**	**53.3**	16.7	3.3
*Smoking status*					
None	755 (95.2)	56.2	23.7	**22.5**	9.1
Current	38 (4.8)	68.4	31.6	**42.1**	18.4
*Physical activity*					
Sedentary	280 (35.3)	**49.6**	26.4	25.0	9.6
Moderately Active	485 (61.2)	**59.8**	23.1	22.5	9.9
Vigorously Active	28 (3.5)	**75.0**	17.9	25.0	3.6
*Daily caffeine intake*					
None	182 (23.0)	**58.8**	18.1	22.0	7.1
1–2	355 (44.8)	**52.1**	25.4	22.5	11.0
3+	256 (32.3)	**61.7**	26.6	25.8	9.4
*Caffeine intake at bedtime*					
None	512 (64.6)	56.3	23.8	22.7	8.4
Any	281 (35.4)	57.7	24.6	24.9	11.7
*Daily alcohol intake*					
None	611 (77.0)	**54.3**	23.4	22.3	10.1
Any	182 (23.0)	**64.8**	26.4	27.5	7.7
*Alcohol intake at bedtime*					
None	701 (88.4)	**55.2**	23.5	23.0	9.6
Any	92 (11.6)	**68.5**	28.5	27.2	9.8
*Total beverages at bedtime*					
None	326 (41.1)	**54.6**	24.2	23.3	8.6
1	287 (36.2)	**52.6**	22.6	19.9	9.1
2+	180 (22.7)	**67.2**	26.1	29.4	12.2

Notes: Bolded values indicate there is a significant association between the covariate and meal timing or sleep at *p* < 0.05 using a chi-squared test.

**Table 2:** Association between meal timing and sleep parameters with and without adjustment for covariates by logistic regression analysis (*N* = 793).

Meal Timing	One or More Nocturnal Awakenings		Sleep Onset Latency >30 min		Sleep Duration ≤7 h	
*Unadjusted model*	**OR**	**95% CI**	OR	95% CI	OR	95% CI
>3 h before bedtime	Ref.	–	Ref.	–	Ref.	–
≤3 h before bedtime	1.61	1.15–2.27	1.24	0.89–1.73	0.79	0.49–1.26
*Adjusted model*	1.43	1.01–2.04	1.10	0.78–1.56	1.03	0.63–1.71

Notes: OR: odds ratio. CI: confidence interval. Unadjusted model with only meal timing entered as a predictor for sleep parameter. Adjusted model for nocturnal awakening included ethnicity and BMI. Adjusted model for sleep onset latency and sleep duration included ethnicity.

## Appendix A. Except from Sleep Patterns, Sleep Environment, Diet, and Exercise Questionnaire.

Responses to the following questions were included in the current study.

### Section 1: Subject Definition

1. What is your age? (years)
2. What is your gender? (M/F)
3. How tall are you? (cm)
4. How much do you weigh? (kg)
5. What is your ethnic background? (Aboriginal/Asian/Caucasian/Indian/Latino/Other—please specify)
6. Do you currently smoke cigarettes? (yes/no)
7. About how many cigarettes do you smoke in a typical day? (1–5/6–10/11–15/16–20/more than a pack a day)

### Section 2: Diet Definition

26. What is the time period between when you typically eat your final meal for the day and when you retire for sleep? (up to 1 h before bedtime/between 1 and 2 h before bedtime/between 2 and 3 h before bedtime/between 3 and 4 h before bedtime/>4 h before bedtime)
28. List the amounts of the following beverages you consume.

	**Daily**	**at Bedtime**
Coffee (cups)		
Decaffeinated coffee (cups)		
Tea (glasses or cups)		
Carbonated drinks (cans/bottles)		
Beer/wine/alcohol (drinks/glasses)		
Milk drinks (cups)		
Chocolate drinks (cups)		
Caffeinated drinks (cans) (e.g., red bull)		

### Section 3: Sleep Experience Definition

30. What sleeping problems have you had? (having trouble falling asleep/waking up once in the middle of the night/waking up more than once each night/waking up 3 or more times each night/early morning wakening)
35. How long does it usually take you to fall asleep? (0–15 min/16–30 min/31–60 min/more than 60 min)
36. What is your usual bedtime? (12–1 a.m./1–2 a.m./2–3 a.m./etc. for all 24 h)
37. What is your usual wake time? (12–1 a.m./1–2 a.m./2–3 a.m./etc. for all 24 h)

### Section 4: Exercise Definition

45. This question is about the time you spent being physically active in the last 7 days.

	**Daily**	**2–3x/week**	**4–5x/week**	**Never**
Low-level exercise (at least 30 min of light physical effort and no noticeable change in breathing, e.g., walking)				
Moderate exercise (at least 20 min of moderate physical effort, making you breathe somewhat harder than normal, e.g., carrying light loads, bicycling at a regular pace, or doubles tennis)				
Vigorous (at least 20 min of hard physical effort and marked rise in heart rate and breathing, e.g., heavy lifting, digging, aerobics, or fast bicycling)				

47. How do you rate yourself? (sedentary, e.g., office worker/moderately active/vigorously active, e.g., building industry)

## Appendix B. Results of Sensitivity Analyses.

Adjustment for caffeine and alcohol at bedtime and adjustment for beverages at bedtime do not make a difference to the association between nocturnal meal timing and sleep. As indicated by the descriptive results, caffeine and alcohol intake were, surprisingly, not significantly associated with the sleep parameters, which indicates that they are not confounders of the relationship between meal timing and sleep. Therefore, adjustment for these variables does not significantly change the results of the study, as demonstrated below.

**Table A1:** Association between meal timing and sleep parameters with and without adjustment for covariates by logistic regression analysis.

Meal Timing	One or More Nocturnal Awakenings		Sleep Onset Latency >30 min		Sleep Duration ≤7 h	
*Model 0*	OR	95% CI	OR	95% CI	OR	95% CI
>3 h before bedtime	Ref.	–	Ref.	–	Ref.	–
≤3 h before bedtime	1.61	1.15–2.27	1.24	0.89–1.73	0.79	0.49–1.26
*Model 1: Adjusted for demographics*	1.43	1.01–2.04	1.10	0.78–1.56	1.03	0.63–1.71
*Model 2a: Adjusted for caffeine and alcohol at bedtime*	1.42	1.00–2.03	1.10	0.78–1.56	1.03	0.62–1.70
*Model 2b: Adjusted for total beverages at bedtime*	1.41	0.99–2.01	1.05	0.74–1.49	1.00	0.60–1.65

Notes: Model 0 is the unadjusted model with only meal timing entered as a predictor for sleep parameter. Model 1 for nocturnal awakening adjusted for ethnicity and BMI. Model 1 for sleep onset latency and sleep duration adjusted for ethnicity only. Model 2a additionally adjusted Model 1 for caffeine and alcohol intake at bedtime. Model 2b additionally adjusted Model 1 for total beverages at bedtime.

Sensitivity analysis was carried out to examine the choice of meal time cut-off point on the observed associations. The results for meal time ≤2 h before bedtime (24.3% of participants) and meal time ≤4 h before bedtime (84.4%) were compared to the results for a meal within 3 h of bedtime. The adjusted results indicate a general reduction in the effect sizes the further away meal times are from bedtime, that is, consistent with the hypothesis that earlier meal times would have less of an influence on sleep. The elevated effect size for nocturnal awakenings remains for meals ≤2 h before bedtime, although this result is not statistically significant. We note that the power to detect significant differences between groups declines as the dichotomised outcome variable moves away from a 50/50 split, which occurs as the meal time cut-off points move away from 3 h.

**Table A2:** Association between meal timing at different cut-off points and sleep parameters with and without adjustment for covariates by logistic regression analysis (*N* = 793).

Meal Timing	One or More Nocturnal Awakenings		Sleep Onset Latency >30 min		Sleep Duration ≤7 h	
***Unadjusted model***	**OR**	**95% CI**	**OR**	**95% CI**	**OR**	**95% CI**
≤2 h before bedtime	1.41	0.98–2.03	1.24	0.85–1.80	1.21	0.71–2.06
≤3 h before bedtime	1.61	1.15–2.27	1.24	0.89–1.73	0.79	0.49–1.26
≤4 h before bedtime	1.57	0.96–2.57	1.33	0.82–2.15	0.61	0.34–1.08
***Adjusted model***						
≤2 h before bedtime	1.40	0.96–2.03	1.16	0.80–1.70	1.43	0.83–2.47
≤3 h before bedtime	1.43	1.01–2.04	1.10	0.78–1.56	1.03	0.63–1.71
≤4 h before bedtime	1.29	0.77–2.16	1.12	0.68–1.84	0.86	0.47–1.58

Notes: Unadjusted model with only meal timing entered as a predictor for sleep parameter. Adjusted model for nocturnal awakening included ethnicity and BMI. Adjusted model for sleep onset latency and sleep duration included ethnicity.
